# Prevention of Diabetic Complications by Walnut Leaf Extract via Changing Aldose Reductase Activity: An Experiment in Diabetic Rat Tissue

**DOI:** 10.1155/2020/8982676

**Published:** 2020-08-15

**Authors:** Zahra Abbasi, Gholamali Jelodar, Bita Geramizadeh

**Affiliations:** ^1^Department of Physiology, School of Veterinary Medicine, Shiraz University, Shiraz 71345, Iran; ^2^Department of Pathology, Shiraz University of Medical Science, Shiraz, Iran

## Abstract

**Background:**

Increased activity of aldose reductase (AR) is one of the mechanisms involved in the development of diabetic complications. Inhibiting AR can be a target to prevent diabetes complications. This study is aimed at evaluating the effect of cyclohexane (CH) and ethanol extracts (ET) of walnut leaves on AR activity in the lens and testis of diabetic rats.

**Methods:**

Fifty-six male rats classified into seven groups as control and treatment groups and treated for 30 days. The treatment groups were treated with different concentrations of ET and CH. The diabetic control (DC) group was exposed to streptozotocin. AR activity was measured in the lens and testis. The expression of AR in the testis was evaluated by the immunohistochemistry method.

**Results:**

Both extracts significantly reduced the AR activity (ng/mg of tissue protein) in the testis (0.034 ± 0.004, 0.038 ± 0.010, and 0.040 ± 0.007 in the treatment groups vs. 0.075 ± 0.007 in the DC group) and lens (1.66 ± 0.09, 2.70 ± 0.47, and 1.77 ± 0.20 in the treatment groups vs. 6.29 ± 0.48 in the DC group) of the treatment group compared to those of the DC group (*P* < 0.05). AR expression in the testes of the treatment groups was decreased compared with that of the DC group (*P* < 0.0001).

**Conclusion:**

Walnut leaf extracts can reduce the activity and localization of AR in the testes and its activity in the lens of diabetic rats.

## 1. Background

Diabetes mellitus is one of the most important endocrine and metabolic disorders and its prevalence increasing globally and expected to rise to approximately 300 million people worldwide until 2025 [1]. Prolonged chronic hyperglycemia is the main cause of complications in diabetes. Male reproductive dysfunction and cataract are important complications of diabetes mellitus [2]. The development of these complications was suggested to be through increasing the activity of aldose reductase (AR) in the polyol pathway (PPW), advanced glycation end products (AGEs), overexpression of AGE receptor, protein kinase C isoform activation, glucosamine pathway activation, and excessive oxidative stress. These conditions can cause important complications such as neuropathy, nephropathy, retinopathy, and cataracts [3]. Typically, less than 3% of serum glucose enters the PPW. However, at high concentrations of glucose, more than 30% of glucose enters this pathway and leads to the accumulation of sorbitol in tissues [4]. Increased oxidative stress after hyperglycemia is caused mainly through autoxidation glycosylation, AGE formation, and increasing polyol pathway activity [5].

Hyperglycemia can evoke a mitochondrial overload in tissues with a high rate of energy metabolism, which results in increased mitochondrial respiratory chain activity and oxidative stress [6].

Hyperglycemia-induced oxidative stress has been proposed as an important factor affecting reproductive function by excessive generation of reactive oxygen species (ROS) that interfere with the ability of the body to neutralize free radicals by either enzymatic or nonenzymatic antioxidants [7]. However, the biochemical structures of the cell membrane impair as ROS possess high affinity polyunsaturated fatty acids [8]. ROS generated in hyperglycemic condition or hypoxic conditions such as varicocele leads to DNA damage, endothelial injury, oxidative stress, apoptosis, and necrosis of germinal cells [9]. Treatment of varicocele-induced rats with hesperidin was reported to improve testicular damage through its antioxidant properties [10].

Lens epithelial cells are the main site of metabolic activity of the lens, and their oxidative damage has an important role in cataract formation [11]. The lens is rich in mitochondria, and only the mature and differentiating fibers as well as epithelium lack mitochondria [12]. Superficial fiber cells and the mitochondria of lens epithelial cells consume 90% of the lens oxygen and are the main sources of endogenous ROS [13, 14]. It is estimated that up to 1% to 5% of oxygen consumed by the lens mitochondria is converted to ROS [15].

Experimental overexpression of AR in the lens of diabetic mice accelerates the development of cataracts [16, 17], whereas knockdown of the AR in rats protects the lens from opacification ex vivo [18]. Previous studies showed that the administration of an AR inhibitor delays the development of cataracts in diabetic dogs and mice [19]. Moreover, several studies have shown that there is a strong relationship between increased AR activity and the risk of other chronic diabetic complications such as neuropathy and retinopathy [20–23].

A decrease in enzymes involved in reducing ROS in the testis tissue of diabetic rats has been reported [24]. Oxidative stress causes cell damage through mechanisms such as destruction in the endothelium of tubule and apoptosis in the testicular germ cell, lipid peroxidation, and DNA and protein oxidative damage in the tissues [25].

Walnut leaves are a good source of antioxidants such as phenol, which can play a crucial role in preventing diseases that involve free radicals as their pathogenesis [26].

Various beneficial effects of the walnut leaf (*Juglans regia L.*) as an antioxidant, anti-inflammatory, and anticancer have already been reported in mice [27–29]. Studies have shown that the use of walnut leaves in the form of aqueous-alcoholic extract, alcohol, cyclohexane, and powder decreases blood glucose level in alloxan- or streptozotocin- (STZ-) induced diabetic rats [30–32]. Oral administration of cyclohexane, ether, and ethanol extracts of walnut leaves was reported to decrease the blood glucose, serum triglyceride, cholesterol, and blood urea nitrogen in rats [32]. Improvement of the activity of sorbitol dehydrogenase in diabetic rats was also reported following treatment with walnut leaf extract [20]. The in vivo antioxidant effects of a polyphenol-rich walnut extract in diabetic animals have frequently been reported [33, 34].

In this study, we aimed to investigate the effect of oral administration of different doses of cyclohexane and ethanol extracts of the walnut leaf on the distribution and activity of aldose reductase in the testis and lens of diabetic male rats.

## 2. Methods

### 2.1. Plant Materials and Extraction

Fresh leaves of *J. regia* were collected from a seedling walnut farm of the Agricultural College of Shiraz University and have been identified and confirmed by a faculty member of the Department of Horticultural Science, Agriculture College, Shiraz University (Prof. M. Rahemi), and a voucher specimen of materials (walnut leaves) has been deposited in the herbarium (No. 96WL14). The leaves were dried in shade, and then milled and soaked in ethanol or cyclohexene for 24 hours. After filtration, the solvent was removed by a vacuum distillation machine and the obtained extract was dried in a lyophilizer. The mentioned method was used to obtain other extracts as the instructions in the previous report [32].

### 2.2. Animals

Fifty-six male Sprague Dawley rats (200 ± 20 g) were procured from the Comparative and Experimental Center of Medical Sciences Department of Shiraz Medical University. Rats were maintained in standard temperature (22 ± 2°C), moisture (38%), and light-dark conditions (12 : 12 h), and standard pellet diet and water were provided ad libitum to the animals.

### 2.3. Animal Ethics

All aspects of animal care and protocols that have been used in this study were in accordance with and approved by the state committee on animal ethics, Shiraz University, Shiraz, Iran. Also, the recommendations of the European Council Directive (86/609/EC) of November 24, 1986, regarding the standards in the protection of animals used for experimental purposes were followed.

#### 2.3.1. Materials

Streptozotocin (N-(methylnitrosocarbamoyl)-*α*-D-glucosamine) was obtained from Sigma Chemical Co., USA, cyclohexane from Merck (KGaA, Germany), ethanol from Haamoon Teb Markazi (Tehran, Iran), Rat Aldose Reductase ELISA Kit from Bioassay Technology Laboratory (Shanghai Crystal Day Biotech Company, LTC, China), and anti-AKR1B1/Aldose Reductase Antibody (aa241-290) IHC plus. All other chemicals and solvents were of analytical grade.

#### 2.3.2. Induction of Diabetes

Diabetes was induced by a single dose of streptozotocin (60 mg/kg body weight, intraperitoneal) in citrate buffer (0.1 M, pH 4.5). 72 hours after STZ injection, by measuring fasting blood glucose levels, diabetes mellitus was confirmed in the animals. Animals with a blood glucose level above 250 mg/dl were included in the diabetic group [35].

### 2.4. Experimental Design

The animals were randomly divided into seven equal groups (*n* = 8), according to the following plan, and treated for 30 days by gavage. The selected doses were according to our previous studies [20, 36].

Three normal healthy groups were treated as follows:

1. Control group: received only vehicle (sesame oil)
2. Treatment control 1: received cyclohexane extract (250 mg/kg/day)
3. Treatment control 2: received ethanol extract (250 mg/kg/day)

Diabetes mellitus was induced in the next four groups as described above and treated as follows:

(4) Diabetic control: diabetic; received the vehicle
(5) Treatment 1: diabetic; received cyclohexane extract (250 mg/kg/day)
(6) Treatment 2: diabetic; received ethanol extract (150 mg/kg/day)
(7) Treatment 3: diabetic; received ethanol extract (250 mg/kg/day)

### 2.5. Evaluation of Aldose Reductase Concentration in the Lens and Testis

On the last day of the experiment, rats were anesthetized by 1.9% diethyl ether-saturated cotton ball in a chamber for 3–5 min and euthanized by whole blood collection through heart puncture. Under the sterile condition, the animal's eye lens was removed and separated from the posterior approach, kept in ice after washing with saline, homogenated in 0.1 M phosphate buffer saline (pH 7.4), and centrifuged at 15000 × g for 30 min at 4°C. The total protein content of the supernatant was determined according to the method described by Bradford [20].

Aldose reductase was determined using a solid-phase sandwich ELISA method (Rat ELISA Kit; Bioassay Technology Laboratory, Shanghai Crystal Day Biotech Company, LTC, China).

### 2.6. Immunohistochemical Evaluation of AR Distribution in the Testis

Immunohistochemical analyses of the tissues were carried out based on protocol described by Kieman (1999). In brief, paraffin-embedded tissue sections (3-4 mm in diameter) were deparaffinized in xylene (three times for ten minutes) and then dehydrated through graded alcohol (100%, 96%, and 70% each of them 20 seconds) and washed with distilled water (2 minutes) and washed with phosphate buffer saline (PBS) (5 minutes). Endogenous peroxidase was inactivated in 3% hydrogen peroxide which stays on the slide for 20 minutes until bubble was observed on the surface of slides and then washed with PBS for 5 minutes. Then, slides were incubated with primary antibody overnight at 4°C and washed with PBS 2 times (each of them 10 minutes) and then incubated with Envision for 20-30 minutes and washed with PBS 2x for 10 minutes Then, slides are incubated in DAB (3,3′-diaminobenzidine) and washed with PBS or distilled water for 5 minutes; the last step is counterstaining with hematoxylin staining and mounting [37].

### 2.7. Statistical Analysis

Results are reported as mean ± SEM. Data were analyzed by SPSS software, version 21.0 for Windows. One-way analysis of variance (ANOVA) followed by post hoc multiple comparisons and Duncan multiple range tests was used to compare mean values between groups, and the significance level was set at *P* < 0.05.

## 3. Results

**Figure 1:**
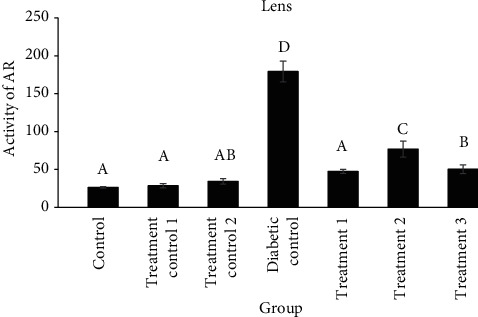
Comparison of aldose reductase (AR) activity (unit/mg of tissue protein) in the lens of different groups (mean ± SEM). Different alphabet letters show significant differences between groups.

**Figure 2:**
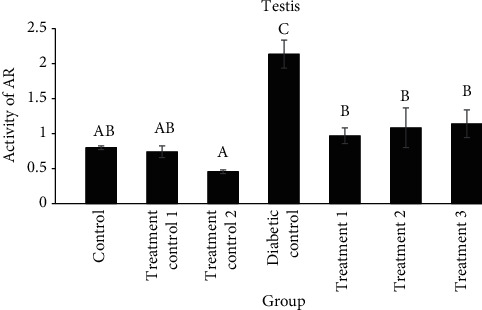
Comparison of aldose reductase (AR) activity (unit/mg of tissue protein) in the testis of different groups (mean ± SEM). Different alphabet letters show significant differences between groups.

The activity of aldose reductase in the lens of diabetic control was significantly higher than that of the treatment groups (*P* < 0.0001). Aldose reductase activity in the testis of the diabetic control group was significantly higher than that of the other groups, and treatment with cyclohexane or ethanol extracts decreased its activity significantly compared to the diabetic control groups (compared to treatment 1 (*P* = 0.0004), treatment 2 (*P* = 0.0032), and treatment 3 (*P* = 0.0026)) (Figures 1 and 2).

### 3.1. Distribution of AR in Rat Testis

**Figure 3:**
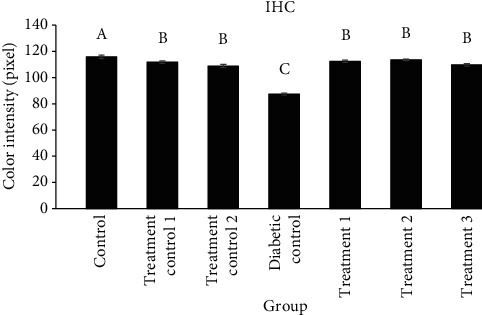
Comparison of the distribution of aldose reductase in the testis in different groups (mean ± SEM). Different alphabet letters show significant differences between groups.

**Figure 4:**
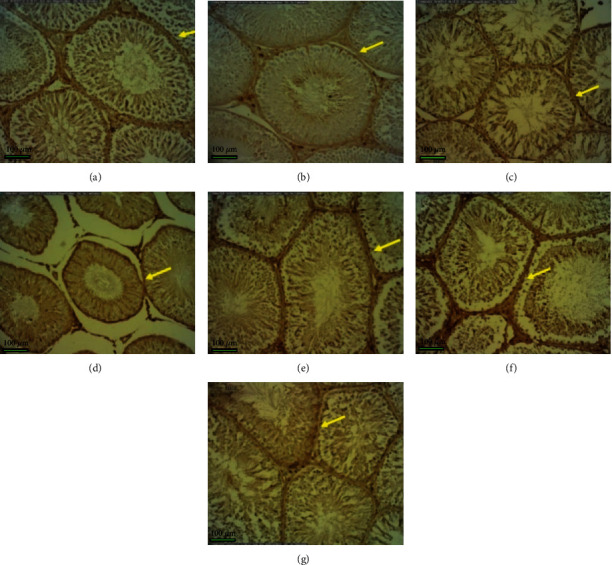
Immunohistochemical localization of aldose reductase in the sections of the testis in different groups: (a) control group; (b) treatment control 1; (c) treatment control 2; (d) diabetic control; (e) treatment 1; (f) treatment 2; (g) treatment 3. Bars = 100 *μ*m. Magnification: ×10.

The distribution of AR in the testis in different groups which was obtained by the immunohistochemical study is shown in Figure 3. The distribution of AR increases significantly in the diabetic control group compared to other groups (*P* < 0.0001), which is shown as a decrease in color intensity in Figures 3 and 4.

## 4. Discussion

Status of glycemic control and the duration of diabetes are important risk factors in the formation of diabetic cataracts [38, 39]. Several hypotheses have been proposed in the pathogenesis of diabetic cataracts, but the activation of PPW and its enzyme AR is of great concern [40].

As was mentioned in Results, walnut leaf extracts decreased the activity and localization of AR in the testes of diabetic rats and also reduced AR activity in the lens of diabetic rats. Plants are rich sources of phytochemicals such as flavonoids and polyphenols which may act as aldose reductase inhibitors [41]. Similar results were reported using cinnamon extract on the activity of aldose reductase of the lens in the in vitro condition and also using Zea mays [41, 42].

Both oxidants and a high level of serum glucose can activate extracellular signal-regulated kinases (ERK) and JNK (c-Jun N-terminal kinases) [43]. ERK activation in the lens may be the cause of the development of cataracts by upregulating GLUT-1, which increases the uptake of glucose for the PPW [44, 45].

It has been reported that hyperglycemia-induced oxidative stresses activate JNK, causing apoptosis in human endothelial cells. JNK activation and hyperglycemia-induced apoptosis were reported to decrease, respectively, by the administration of vitamin C (as an antioxidant) [46].

Oxidative stress and mitochondrial dysfunction are important features of metabolic disorders, and hyperglycemia can evoke a mitochondrial overload in tissues with a high rate of energy metabolism, resulting in increased mitochondrial respiratory chain activity and oxidative stress [6].

During hyperglycemia, the balance between production and elimination of free radicals is also disrupted. As a result, free radicals increase and cause oxidative stress. Oxidative stress causes cell damage through mechanisms such as lipid peroxidation and DNA and protein oxidative damage in the tissues [47]. Moreover, oxidative species promote the glycoxidation reaction of proteins in the presence of reducing sugars, thus resulting in AGE accumulation.

It has been proven that the PPW is the primary mediator of diabetes-induced oxidative stress in the lens [48]. Researchers have focused on the first step of the PPW as initiating factors in diabetic cataract formation, which occur due to the accumulation of sorbitol-induced stress as osmotic stress in the endoplasmic reticulum (ER), the main site of protein synthesis, leading to the generation of free radicals. The stress of ER may be due to glucose level disturbance initiating an unfolded protein response which causes oxidative stress damage to lens fibers [49, 50].

A high level of glucose in the aqueous humor may cause glycation of lens protein, resulting in the formation of superoxide radicals (O^−2^) [51].

It has been shown that in diabetic rats, flavonoids such as quercetin or isoflavone genistein delayed the formation of diabetic cataracts [52, 53]. Also, the treatment of diabetic rats with ginger significantly inhibited the formation of various AGE products, including carboxymethyl lysine in the lens, and delayed the progression and onset of cataract [54]. Moreover, a combination of vitamin E, a lipid-soluble and antioxidant vitamin, and insulin could prevent the formation and progression of cataracts in the animals [55].

The walnut leaf extract contains ingredients such as quercetin, kaempferol, eugenol, avicularin, nicotine, caffeic acid, hyperin, beta-eudesmol, juglone, p-Coumaric acid, ascorbic acid, ellagic acid, gallic acid, neochlorogenic acid, and cyaniding, while quercetin and kaempferol are possibly major active ingredients [56, 57].

Although the PPW causes oxidative stress in both the lens and the nerve, its role in the development of diabetic lesions in these two tissues seems to be different. The activation of AR in the PPW can lead to the alteration of various metabolic factors, particularly the formation of ROS which leads to oxidative stress, as the initial and main inducing factor of cataract [16].

The present study showed that oral administration of different doses of cyclohexane and ethanol extracts of the walnut leaf decreases the activity of AR in all of the diabetic treated rats compared with the diabetic control group significantly. This reduction was more obvious in the lens of diabetic treatment 1 compared to diabetic treatments 2 and 3. It can be suggested that the action of antioxidants present in the walnut leaves [36] improves the activity of AR in the diabetic groups. The results of this study are consistent with the results obtained by Yoshida et al. and Goodarzi et al. [55, 58], and it can be concluded that the antioxidants and quercetin in the walnut leaf may be effective in reducing the activity of the aldose reductase. A decrease in the activity of AR in the lens was also reported using cinnamon extract in in vitro condition [41].

Our results showed that oral administration of different doses of cyclohexane and ethanol extracts in the diabetic groups decreases the aldose reductase activity and its distribution in the testicular tissue compared to the diabetic control group.

Hyperglycemia-induced oxidative stress has been proposed as an important factor affecting reproductive function by excessive generation of reactive oxygen species (ROS). A decrease in enzymes involved in reducing ROS in the testis tissue of diabetic rats has been reported, and treatment with *Galega officinalis* extract was reported to improve oxidative stress and tissue damage [24]. Oxidative stress causes cell damage through mechanisms such as destruction in the endothelium of tubule and apoptosis in the testicular germ cell, lipid peroxidation, and DNA and protein oxidative damage in the tissues [25].

Because of the high concentration of steroid in the male reproductive system, it is exposed to oxidative stress caused by high metabolism activity, which results in a high concentration of reducing sugars [59, 60]. At a particular stage, increased cell death in germ cells has been observed in the seminiferous tubules of diabetic animals [61].

It has been suggested that the strong antioxidant activity of flavonoids present in plants can play a protective role against diseases caused by oxidative stress, and recent attention has focused on the use of flavonoids to prevent and treat the diseases [62, 63]. Therefore, the effect of walnut leaf extracts on the distribution of aldose reductase in testicular tissue could be due to its flavonoid's effect on the PPW route and ultimately on oxidative stress.

Considering the inhibitory effects of other plants such as tea leaves [64], Zingiber officinale [65], and Phyllostachys nigra [66] on aldose reductase, the antioxidant properties of walnut leaves, and our finding regarding the decrease of aldose reductase activity and distribution in the testicular tissue of the treatment groups, it can be proposed that the flavonoid antioxidants of walnut leaves can reduce diabetic oxidative stress through alteration of the polyol pathway, which may prevent or delay some of the important complications of diabetes mellitus. This finding may confirm other reports for the use of antidiabetic plants to ameliorate complications in diabetic patients.

## 5. Conclusion

It can be concluded that walnut leaf extracts can reduce the activity and localization of AR in the testes of diabetic rats and also reduce AR activity in the lens of diabetic rats. This finding was confirmed by the evaluation of enzyme activity in the lens and testis and its distribution in the testis by immunohistochemical study. However, further studies are needed to establish these effects in other species or doses to be applicable in human beings.

### 5.1. Study Limitations and Suggestions

Other biological methods to evaluate the expression of AR and SDH in the tissue are useful, but due to budget limitation, we were not able to do so in the current study.

## Data Availability

All the data generated in this current work are included in Results and Discussion. Raw data supporting the findings of the current work are available from the corresponding author on reasonable request. A voucher specimen of walnut leaves has been deposited in the herbarium.
